# Three-dimensional evaluation of upper airway changes following rapid maxillary expansion: A retrospective comparison with propensity score matched controls

**DOI:** 10.1371/journal.pone.0261579

**Published:** 2021-12-23

**Authors:** Hussein Aljawad, Kyung-Min Lee, Hoi-Jeong Lim

**Affiliations:** 1 Department of Orthodontics, School of Dentistry, Chonnam National University, Gwangju, Korea; 2 Department of Orthodontics and Dental Education, School of Dentistry, Chonnam National University, Dental Science Research Institute, Gwangju, Korea; International Medical University, MALAYSIA

## Abstract

**Background:**

The aim of this study is to evaluate upper airway changes three-dimensionally following rapid maxillary expansion (RME) and compare the changes with matched controls.

**Materials and methods:**

Seventeen patients (mean age 12.6 ± 1.8 years) with maxillary transverse deficiency were treated with RME. Using the propensity score matching method, 17 patients (mean age 12.3 ± 1.5 years) were selected from a non-RME control group of 33. Case-control matching was performed based on 5 covariates: age, gender, CBCT scan interval, sagittal skeletal pattern, and tongue posture. Airway volumes of nasopharynx and oropharynx and minimum cross-sectional areas (MCA) of oropharynx were measured and compared between the case and control groups in CBCT scan images.

**Results:**

In the case group, significant increases from before to after RME were found in all measurements except MCA of the retroglossal segment of oropharynx. Before treatment, there were no significant differences between case group and control group. While comparing the case group with the control group after treatment showed overall greater increases in the case group. In particular, MCA of retropalatal segment showed statistically significant differences.

**Conclusion:**

The results of this study indicate that RME causes an increase in upper airway dimensions.

## Introduction

Rapid maxillary expansion (RME) is a common technique used to correct maxillary transverse deficiencies and posterior crossbites in young patients. The effect of RME on upper airway has been evaluated using various methods including polysomnography, cephalometrics, fluid dynamics, rhinomanometry, and acoustic rhinometry [1–4]. Functional evaluation of upper airway showed that treatment with RME significantly improves breathing functions [5].

Upper airway dimensions following RME treatment have been also evaluated using cone-beam computed tomography (CBCT); however, only a few studies have compared their case group with a control group [6–13]. The studies that have compared their case group with a control group showed inconsistent results [11–13]. For example, while Iwasaki et al. [12] reported that the upper airway volumes increased significantly following RME treatment when compared with a control group, Zhao et al. [11] demonstrated that RME did not cause significant change in oropharyngeal airway volumes.

As hinted, there has been much controversy over the effect of RME treatment on upper airway dimensions. To effectively analyze the effect of RME while controlling for confounding factors and the inconsistency, this study utilizes the propensity score matching (PSM) method to select the control group. PSM is a statistical method designed to evaluate the treatment effect in situations where Randomized Controlled Trials are difficult to perform [14, 15]. It is widely used in clinical studies because it can adjust for the pretreatment differences and, therefore, reduce selection bias and enable randomization. Furthermore, PSM is a data reduction method that uses one propensity score rather than multiple covariates; in other words, it is a more appropriate method for small samples sizes, including this study.

The purpose of this study is to evaluate upper airway changes three-dimensionally following RME and to compare the case and control group, which was selected based on the five covariates: age, gender, CBCT scan interval, sagittal skeletal pattern, and tongue posture using PSM.

## Materials and methods

**Fig 1:**
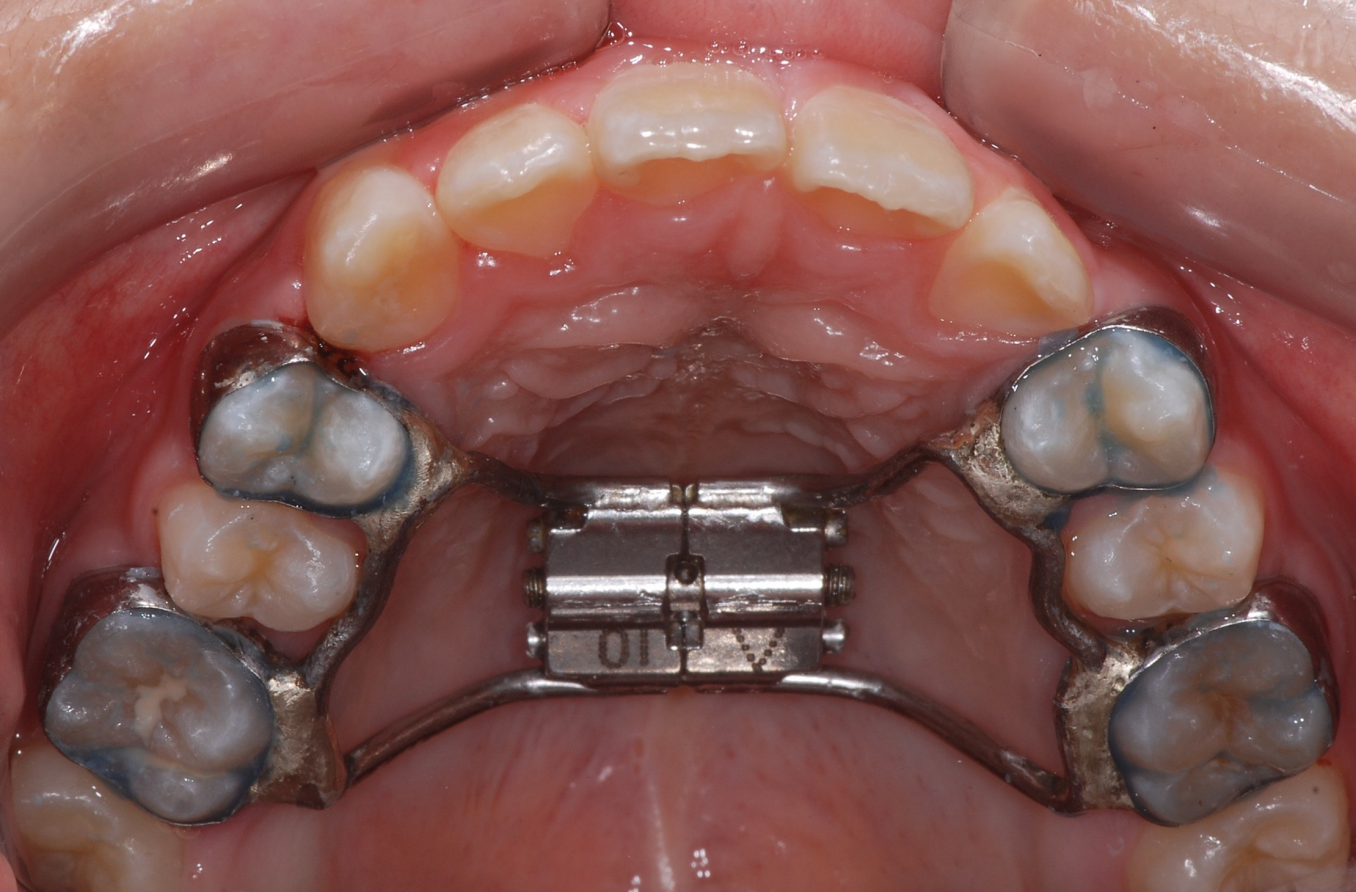
Rapid maxillary expansion. Intraoral photo of rapid maxillary expansion.

This study was approved by the Institutional Review Board of Chonnam University Dental Hospital #CNUDH-2017-009. It is a case-control retrospective study designed to evaluate the changes in upper airway dimensions following RME. In this study, the data for the case and control subjects were collected from the hospital database. The case group included subjects who had undergone RME (Fig 1) as part of their comprehensive orthodontic treatment. The inclusion criteria were as follows: patients between 10–16 years of age, treated with Hyrax palatal expander (Dentaurum, Ispringen, Germany), diagnosed with maxillary transverse deficiency, had an initial CBCT scan before RME treatment (T0) and a progress CBCT scan after at least 4 months of retention following expansion (T1). The control group included patients who underwent orthodontic treatment without RME. Subjects with previous orthodontic treatment, respiratory diseases, enlarged tonsils, or history of adenoidectomy, tonsillectomy, systemic disease, or craniofacial anomalies were excluded.

Subjects who had been treated with activator or bionator functional appliances were also excluded due to the potential of these appliances to enhance upper airway dimensions [16–18]. Seventeen and thirty-three subjects were identified for the case and control groups, respectively.

The case group was treated with Hyrax palatal expander fixed to the first permanent premolars and first permanent molars. In cases of unerupted permanent first premolars, the primary second molars were banded instead. The activation protocol was to turn the screw twice per day until the palatal cusps of the maxillary molars contacted the buccal cusps of the mandibular molars. Then RME screws were fixed with resin and kept in place no less than 3 months for retention.

Propensity score matching (PSM) analysis was used for one-to-one matching between case and control groups. Unlike one-to-one matching based on age and gender only, PSM has the advantage that two groups can be one-to-one matched using multiple covariates to reduce selection bias in non-randomized studies [14, 15]. PSM analysis were performed according to five covariates including age, gender, CBCT scan interval, sagittal skeletal pattern, and tongue posture. Seventeen control subjects were one-to-one matched to case subjects based on the previously mentioned covariates.

CBCT scans were obtained with Alphard Vega (Asahi Roentgen Co., Kyoto, Japan) under the following conditions: 80 kV, 5 mA, 0.39×0.39×0.39 mm voxel size, and 200×179 mm field of view. CBCT scans were taken before RME insertion (T0) and at least 4 (mean 10.5±5.3) months after the last screw activation (T1). Patients were scanned in upright position with teeth in occlusion using reference ear plugs and head posture aligner to be used later for orienting the CBCT scans in Invivo5 software [19]. None of the CBCT scans were ordered for research purposes as all CBCT data were already available in the hospital database. The individual in this manuscript has given written informed consent to publish these case details.

Low tongue posture was identified on the patients’ CBCT images. On the sagittal view of the CBCT image, the position of the tongue was evaluated. The physiological resting position of the tongue was defined when the tongue is in contact with the palate and extending to the palatal aspect of the alveolar ridge [20, 21]. Patients with tongue posture that is not in accordance with the definition of physiological resting position were identified as patients with low tongue posture. In the sagittal view, if the tongue dorsum did not touch the palate and the palatal aspect of the alveolar ridge, and the superior border of the tongue was traced clearly, the patient was considered to be a low tongue posture patient.

Initial (T0) CBCT images were first imported into Invivo5 software (version 5.3, Anatomage, San Jose, California) and oriented using the reference ear plugs and head posture aligner. The T1 CBCT images were superimposed over the initial CBCT images (T0) and saved after superimposition. Then, using the *import orientation* function of the program, the T1 CBCT images were oriented in the same position with T0 CBCT images when measuring airway dimensions.

**Fig 2:**
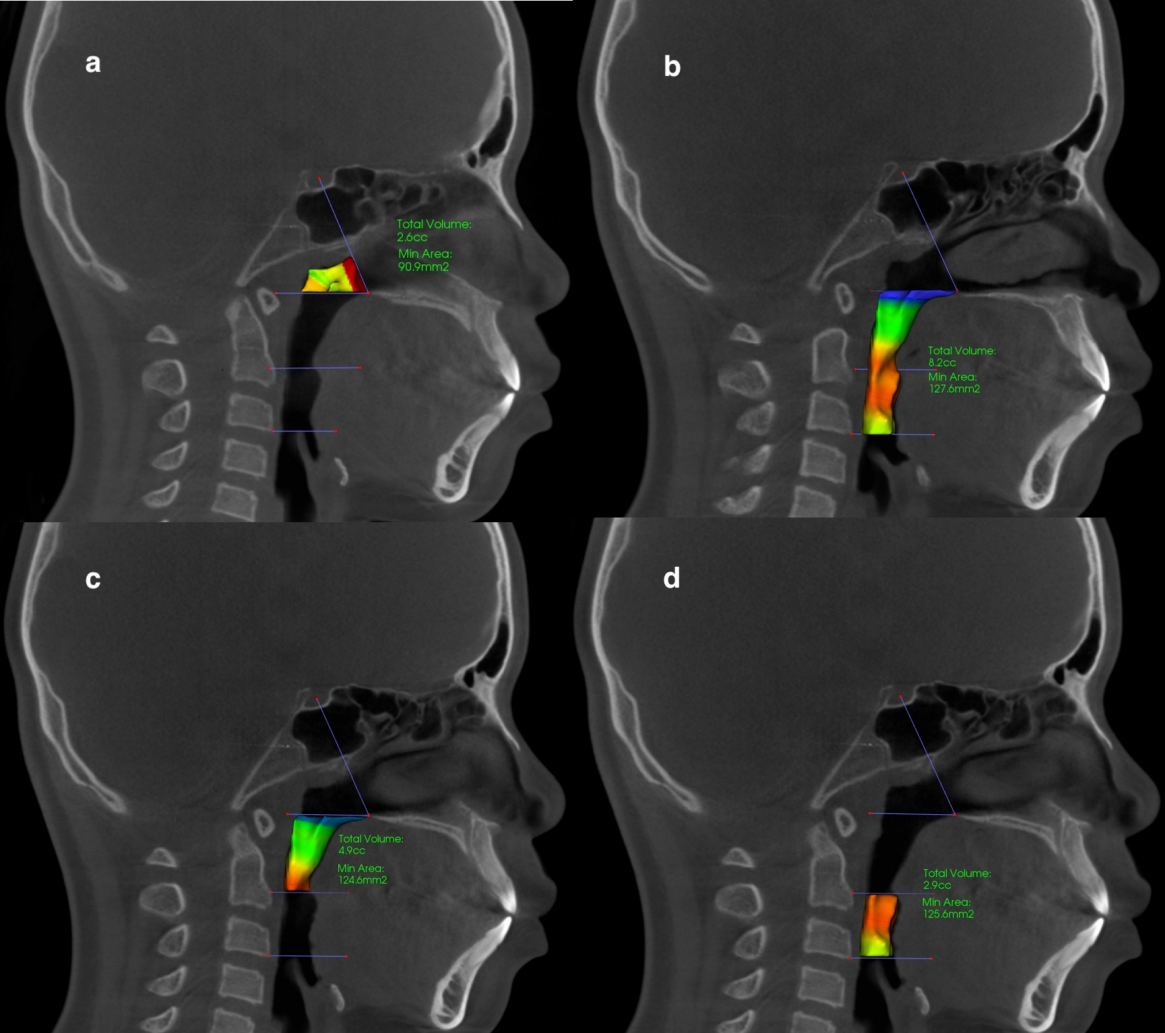
Upper airway segments. (a) nasopharyngeal airway (b) oropharyngeal airway (c) retropalatal segment of the oropharyngeal airway (d) retroglossal segment of the oropharyngeal airway.

In case and control groups, the oropharyngeal airway volume was defined as the volume between 2 planes; the superior plane, defined on the midsagittal image as a horizontal line passing through the posterior nasal spine (PNS) parallel to the floor (provided by the software), and the inferior plane, defined as a horizontal line passing through the most anteroinferior point of the third cervical vertebrae. Oropharynx was further divided into retropalatal and retroglossal segments by a plane passing from the most inferior point of uvula parallel to the floor (Fig 2).

Nasopharyngeal airway volume was limited superiorly by a plane passing through the sella (S) and the PNS perpendicular to the sagittal plane. The inferior border of nasopharynx was the superior border of the oropharynx. The minimum cross-sectional area (MCA) was also calculated for the retropalatal and retroglossal airway segments. To evaluate the amount of transverse expansion, the intermolar and interpremolar distances in case and control groups were measured before and after treatment.

### Statistical analysis

Sample size calculation was performed using G*power program (version 3.1.9.2; Germany) with effect size of 0.73 from a pilot study at 5% significant level and 80% power. The power analysis showed that minimum sample size should be of 17 patients. Therefore, 17 patients were collected form the dental hospital database. PSM analysis performed for one-to-one matching of case and control groups using R software (Version 3.3; The R Foundation for Statistical Computing). An optimal matching technique was used to reduce the average absolute distance across all pairs. It also ensured that all case subjects were one-to-one matched. Histograms and jitter plot were drawn to check the distribution of propensity scores before and after matching for case and control group.

The differences in characteristics between case and control groups before and after matching were evaluated. An independent *t*-test was used for age and T0-T1 CBCT scan interval covariates before matching, while a paired *t*-test was used for the same covariates after matching. A chi-square test was used for gender, sagittal skeletal pattern, and tongue posture before matching. A McNemar test for paired dichotomous data was used for gender and tongue posture after performing matching analysis. An extended McNemar test was used for the sagittal skeletal pattern following matching, as it is a 3 × 2 table. A paired *t*-test was used to evaluate the changes in upper airway dimensions. Statistical evaluations were performed at 0.05 significance level.

## Results

Intraclass correlations were performed between the first and second measurement within 2-weeks interval and it ranged between 0.957–0.971. The inter-rater reliability ranged between 0.92–0.96 which indicated that the reliability of the measurements was high. The intermolar and interpremolar distances in the case group were expanded by 5.9±2.9 mm and 5.6±2.7 mm, compared with 0.7±0.8 mm and -0.1±1.2 mm in the control group, respectively. None of the characteristics between the case and control groups before and after matching showed statistical significance (Table 1).

**Table 1:** Characteristics of case and control group before and after matching.

	Before matching			After matching		
	Case group (N = 17)	Control group (N = 33)	*P*-value	Case group (N = 17)	Control group (N = 17)	*P*-value
**Age (years)**	12.6±1.8	13.0±1.8	0.407*	12.6±1.8	12.3±1.5	0.512^†^
**Gender**						
** Male**	3	7	1.000^§^	3	4	1.000^‖^
** Female**	14	26		14	13	
**CBCT scan Interval**	10.5±5.3	11.0±4.8	0.757*	10.5±5.3	11.5+5.3	0.540^†^
**(months)**						
**Skeletal pattern**						
**Class I**	9	15	0.450^‡^	9	11	0.881^¶^
**Class II**	2	9		2	2	
**Class III**	6	9		6	4	
**Low Tongue posture**						
** With**	4	10	0.613^‡^	4	5	1.000^‖^
** Without**	13	23		13	12	

*Independent *t*-test

^†^paired *t*-test

^‡^chi-square test

^§^Fisher exact test

^‖^McNemar’s test

^¶^extended McNemar’s test.

In the matched control group, none of the airway volumes and MCAs showed significant change between initial and progress CBCT scans. The retroglossal airway volume, and the MCAs of the retropalatal and retroglossal airways in the control group showed insignificant decrease between initial and progress CBCT scans. Unlike the matched control group, nasopharyngeal, retropalatal and retroglossal airway volumes were significantly increased in the case group following treatment with RME. The MCA of the retropalatal airway were also significantly increased (*P* = 0.007) following treatment with RME. The MCA of retroglossal airway volume were also increased following treatment with RME in the case group, however, did not reach statistical significance (Table 2).

**Table 2:** Comparison of airway parameters between T0 and T1 for the case group (N = 17) and control group (17).

Variable	Control Group				Case Group			
	Initial	Progress	Difference	Significance	Before RME	After RME	Difference	Significance
	Mean ± SD	Mean ± SD	Mean ± SD	*P*-value	Mean ± SD	Mean ± SD	Mean ± SD	*P*-value
**Nasopharynx**								
**Volume (mm**^**3**^**)**	2782 ± 1479	3094 ± 1130	311 ± 868	0.158	3123 ± 1310	3782 ± 1690	658 ± 1029	0.018*
**Retropalatal segment of oropharynx**								
**Volume (mm**^**3**^**)**	4164 ± 2046	4423 ± 1992	258 ± 970	0.288	4758 ± 1517	5629 ± 1915	871 ± 1170	0.007*
**MCA**^**†**^ **(mm**^**2**^**)**	124.76 ± 78.41	117.44 ± 75.63	-7.32 ± 47.98	0.538	137.92 ± 45.14	172.39 ± 69.75	34.46 ± 46.28	0.007*
**Retroglossal segment of oropharynx**								
**Volume (mm**^**3**^**)**	4141 ± 2287	4088 ± 2536	-52 ± 1642	0.896	4800 ± 2028	5788 ± 3296	988 ± 1.746	0.033*
**MCA**^**†**^ **(mm**^**2**^**)**	134.48 ± 74.0	126.22 ± 71.27	-8.26 ± 50.25	0.507	149.65 ± 48.51	172.37 ± 68.87	22.72 ± 45.08	0.102

*P < 0.05 on paired *t*-test

^†^MCA, minimum cross-sectional area

The comparison of nasopharyngeal, retropalatal and retroglossal airway volumes between the case group and control group before treatment showed insignificant differences. The differences in the MCAs of the retropalatal and retroglossal airways between the case and control group before treatment were also insignificant. After treatment, the case group showed an overall greater airway volumes and MCAs compared to the matched control group. However, only the MCA of the retropalatal airway showed significant difference (*P* = 0.038) between the case and control group and the MCA of the retroglossal airway almost reached statistical significance (p = 0.065) (Table 3).

**Table 3:** Comparison of airway parameters between case group (N = 17) and control (N = 17) group before and after treatment.

Variable	Before				After			
	Case group	Control group	Difference	Significance	Case group	Control group	Difference	Significance
	Mean ± SD	Mean ± SD	Mean ± SD	*P*-value	Mean ± SD	Mean ± SD	Mean ± SD	*P*-value
**Nasopharynx**								
**Volume (mm**^**3**^**)**	3123 ± 1310	2782 ± 1479	341 ± 1760	0.436	3782 ± 1691	3094 ± 1131	0.69 ± 1.53	0.083
**Retropalatal segment of oropharynx**								
**Volume (mm**^**3**^**)**	4758 ±1517	4164 ± 2046	594 ± 2697	0.377	5629 ± 1915	4424 ±1993	1206 ± 2846	0.100
**MCA**^**†**^ **(mm**^**2**^**)**	137.92 ± 45.14	124.76 ± 78.41	13.16 ± 93.08	0.568	172.39 ± 69.75	117.44 ± 75.63	54.95 ± 100.40	0.038*
**Retroglossal segment of oropharynx**								
**Volume (mm**^**3**^**)**	4800 ± 2028	4141 ±2287	659 ± 2798	0.346	5788 ± 3296	4088 ± 2536	1700 ± 4053	0.103
**MCA**^**†**^ **(mm**^**2**^**)**	149.65 ± 48.51	134.48 ± 74.0	15.17 ±91.79	0.505	172.38 ± 68.87	126.22 ± 71.27	46.16 ± 96.08	0.065

*P < 0.05 on paired *t*-test

^†^MCA, minimum cross-sectional area

The comparison of airway volumes changes following treatment between the case group and control group showed that the increase in nasopharyngeal, retropalatal and retroglossal airway volumes were greater in the case group (658 mm^3^, 870 mm^3^ and 988 mm^3^ respectively) than in the control group (311 mm^3^, 258 mm^3^ and -52 mm^3^ respectively). The change in the MCAs of the retropalatal and retroglossal airway following treatment were also greater in the case group (34.46 mm^2^ and 22.72 mm^2^ respectively) than in the control group (-7.32 mm^2^ and -8.26 mm^2^ respectively). Even though there were overall greater changes in the case group compared to the control group, only the MCA of the retropalatal airway show significant increase (*P* = 0.021) (Table 4).

**Table 4:** Comparison of airway parameters changes between case group (N = 17) and control group (N = 17).

Variable	Case group	Control group	Difference	Significance
	Mean ± SD	Mean ± SD	Mean ± SD	*P*-value
**Nasopharynx**				
**Volume (mm**^**3**^**)**	658 ± 1028	311 ± 868	347 ± 1671	0.405
**Retropalatal segment of oropharynx**				
**Volume (mm**^**3**^**)**	870 ± 1169	258 ± 970	611 ± 1569	0.128
**MCA**^**†**^ **(mm**^**2**^**)**	34.46 ± 46.28	-7.32 ± 47.98	41.78 ± 67.08	0.021*
**Retroglossal segment of oropharynx**				
**Volume (mm**^**3**^**)**	988 ± 1745	-52 ± 1642	1041 ± 2527	0.109
**MCA**^**†**^ **(mm**^**2**^**)**	22.72 ± 54.08	-8.26 ± 50.25	30.99 ± 77.60	0.119

*P < 0.05 on paired *t*-test

^†^MCA, minimum cross-sectional area

**Fig 3:**
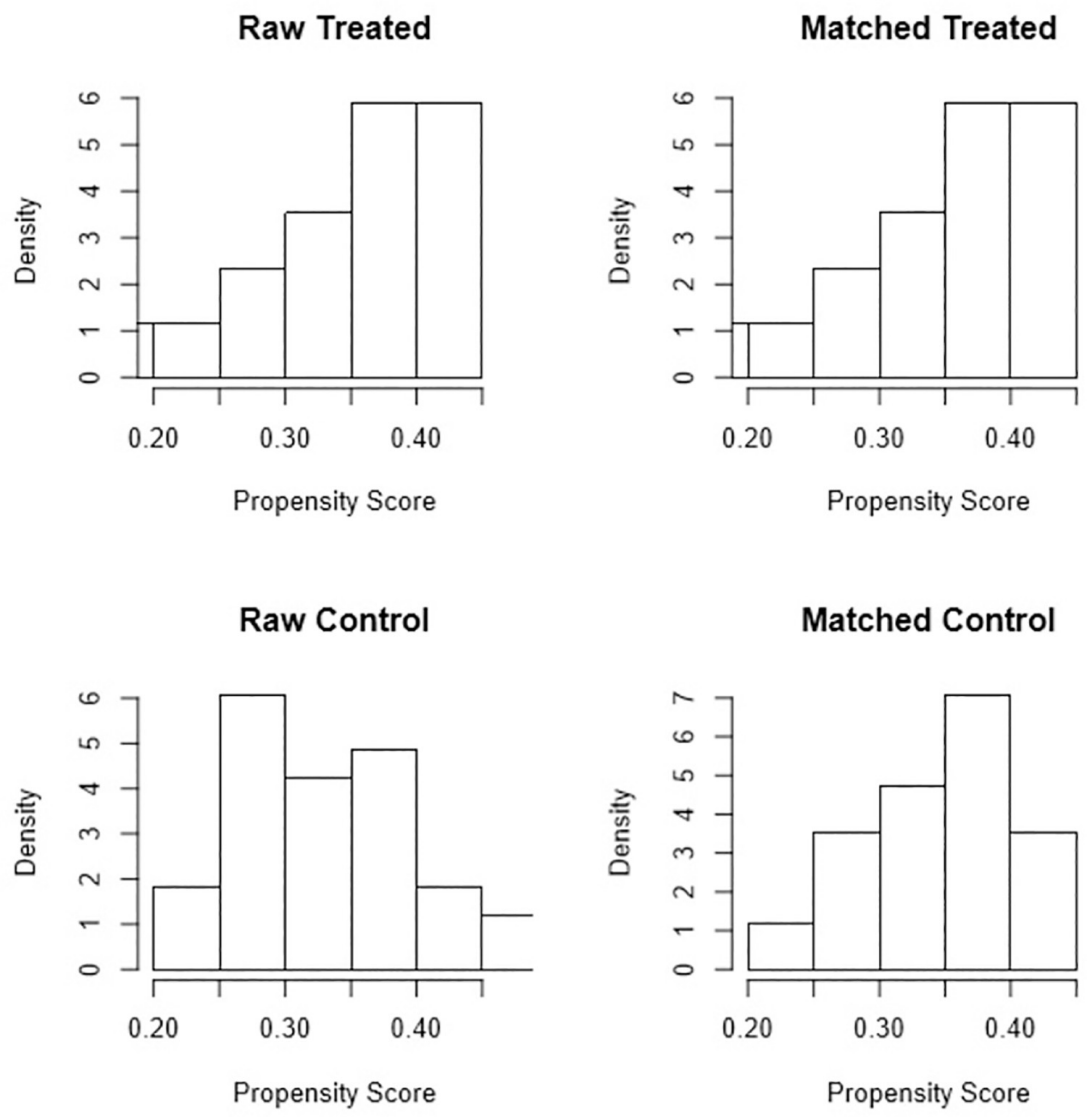
Histogram plot. Histograms showing the density of propensity score distribution in the treated and control groups before and after matching.

**Fig 4:**
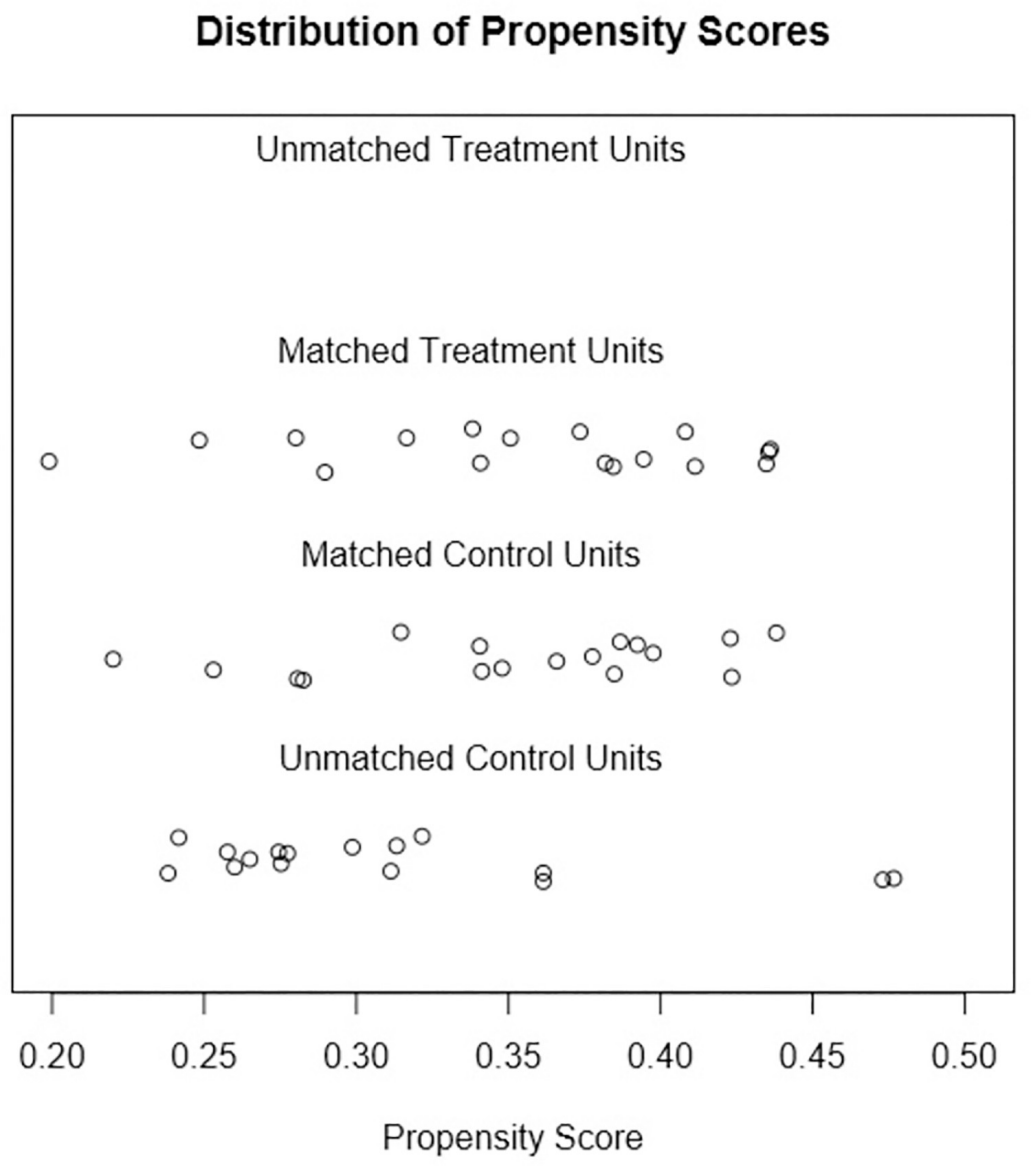
Jitter plot. Jitter plot of propensity scores before and after treatment for case and control group.

The distributions of propensity scores for the case and control groups before and after matching were shown in Fig 3. It was found that the histograms of the matched RME and control groups looked more similar than before matching. The jitter plot in Fig 4 showed that each subject from the case group were matched with a subject from the control group. It also showed that the unmatched control subjects were disregarded.

## Discussion

The main objective of this study was to evaluate upper airway changes three-dimensionally following RME and compare the changes with a matched control group. In literature, several studies have evaluated nasopharyngeal and oropharyngeal airway changes following RME using CBCT [6–13]. However, few of them compared the changes in the upper airway between case and control groups [11–13]. In the current study, potential confounding variables that might affect nasopharyngeal and oropharyngeal airway dimensions were included as covariates when selecting a one-to-one matched control sample.

Schendel et al. has showed that airway volume increases until the age of 20 [22]. Therefore, Age and CBCT scan interval were used as matching covariates to exclude growth as a factor for change in upper airway volumes and MCA. Gender was included in the matching covariates as it has been demonstrated that there are structural and functional differences in upper airway between men and women [23]. The sagittal skeletal pattern was also used due to the strong relationship between the anteroposterior skeletal pattern and airway volume and MCA [24]. Tongue posture before orthodontic treatment, which might affect upper airway morphology and dimensions, was also used as a covariate [25–27]. Use of several matching variables in addition to age and gender increases the difficulty of one-to-one matching of case and control subjects in observational studies. Therefore, PSM analysis was proposed for this study. PSM analysis helps in matching case and control groups based on multiple covariates. In addition, PSM attempts to mimic randomization.

EI et al. evaluated the changes in the oropharyngeal airway volume following treatment with RME [13]. They reported that oropharyngeal airway volume increased significantly following treatment with RME which is consistent with the results of this study. However, when compared with a control group, EI et al. reported that the change in oropharyngeal airway volume was insignificant [13]. Chang et al. reported that there were no significant changes in oropharyngeal, retropalatal, or retroglossal airway volumes following RME [7]. However, they reported that the cross-sectional area between the PNS and basion showed a significant increase in RME patients. In the current study, the upper airway volumes were increased significantly following treatment with RME in the case group, however only the MCA of the retropalatal segment of oropharynx showed significance when compared with a matched control group. Chen et al. [28] has demonstrated that narrowing of the retropalatal airway is the major contributor of upper airway obstruction in Asian patients with obstructive sleep apnea. The current study showed that when compared with a matched control group, RME treatment caused significant change in the MCA of the retropalatal airway and almost reached statistical significance in the MCA of the retroglossal airway (*P* = 0.065).

Smith et al. [6] also evaluated upper airway volumes three-dimensionally before and after RME treatment. Unlike our results in the case group, they concluded that RME did not induce a significant change in retropalatal and retroglossal airway volumes. However, they also concluded that RME induces significant increase in nasopharyngeal volume, which is consistent with the current study. Iwasaki et al. [12] noted that the drawback of previous studies that evaluated upper airway volume following RME is that they did not control tongue posture when obtaining CBCT. Tongue posture is an important factor that can affect upper airway shape and size. In their study, they evaluated the change in tongue posture and upper airway following RME. They reported that total upper airway volumes increased significantly following RME compared to the control group. They also indirectly evaluated the change in tongue posture relative to the palate by measuring the intraoral airway volume before and after RME treatment. They reported that intraoral airway volume decreased significantly following RME, indicating that the low tongue posture became higher following treatment with RME. In the current study, tongue posture before RME was considered a confounding variable and was used as a covariate. The tongue posture was not evaluated following RME and was only evaluated before RME for matching purposes. It was meant to adjust for error that tongue posture might have on upper airway before starting the RME treatment. The number of patients with low tongue posture became the same in the case and control groups following matching.

In Randomized Control Trials, study subjects are randomized so that there are no differences in characteristics that affect the results. However, randomization in Randomized Control Trials might cause ethical problems. On the other hand, observational studies are conducted on a specific population without randomization, which makes it impossible to infer causal relationships because selection bias cannot be avoided when selecting study subjects [29].

For subjects with low incidence, simple one-to-one matching technique makes it difficult to collect enough sample size. In addition, simple one-to-one matching technique might produce distorted results due to small number of matching covariates that could be used. On the other hand, multiple regression analysis is the method to use when there are many covariates. However, the sample size of multiple regression method should be 10 to 20 times as big as the number of covariates [30]. Unlike regression analysis, PSM analysis can achieve reliable results even in studies with small sample sizes. Hence, PSM analysis is more appropriate to use than regression analysis to find out the therapeutic effect in studies with small sample sizes [31]. PSM creates the matching control group by calculating the propensity scores that reflect the influence of the covariates on the case and control groups. In addition, PSM analysis is used in studies where randomization is difficult. In dentistry, where there are many studies of small sample size, the approach of the PSM method could be of useful.

## Conclusions

The results of this study demonstrated that the case group showed significant increases in upper airway dimensions following RME whereas the matched control group did not show significant changes in upper airway dimensions. The comparison of upper airway dimensions changes following treatment between the case group and the matched control group showed an overall increase in upper airway dimensions of the case group as compared to the matched control group but with insignificant trend. However, the MCA of the oropharynx showed significant increase.
